# Whole-genome sequence of *Periconia* sp. strain TS-2, an ascomycete fungus isolated from a freshwater outflow and capable of Mn(II) oxidation

**DOI:** 10.1128/MRA.00599-23

**Published:** 2023-11-06

**Authors:** Shihori Tsushima, Robert A. Kanaly, Jiro F. Mori

**Affiliations:** 1 Graduate School of Nanobioscience, Yokohama City University, Yokohama, Japan; University of California, Riverside, Riverside, California, USA

**Keywords:** manganese-oxidizing fungi, genome sequence, *Periconia*

## Abstract

Members of the genus *Periconia* are commonly found as plant-associated filamentous fungi. Here, the first draft genome sequence of a new *Periconia* strain, TS-2, that was isolated from freshwater outflow sediment and possesses the ability to oxidize dissolved Mn(II), was obtained and has an estimated size of 40.7 Mb.

## ANNOUNCEMENT

A new isolate of the fungus *Periconia* (*Ascomycota*, *Dothideomycetes*, *Pleosporales*), strain TS-2, was obtained from the sediment of a natural groundwater outflow in Sakae-ku, Yokohama, Japan, that contained 1.5 mg/L dissolved Mn(II) (by the periodate oxidation method) when the sampling was conducted in May 2021. Through the screening of microorganisms for Mn(II) oxidation capabilities by using a medium which consisted of 40 mg/L MnSO_4_•5H_2_O, 50 mg/L yeast extract, and 2% agarose (1), strain TS-2 was obtained as a mycelial colony that accumulated brownish Mn-oxide precipitates and was purified. Members of the fungal genus *Periconia* have been characterized as endophytic fungi known to produce pharmacologically valuable natural products (2–4), and Mn(II) oxidation in *Periconia* has rarely been investigated; Mn(II) oxidation was first reported in *Periconia* in 2023 in strain SM10a2_F1 (5), and the genome of this organism is not sequenced. Consequently, the genome of strain TS-2 was sequenced, and the functional genes were investigated to expand our understanding of the physiology of this fungal genus.

To extract genomic DNA, the mycelium of strain TS-2 that was cultured for 9 days on potato dextrose agar was subjected to cell lysis by adding Lysis Solution F (Nippon Gene, Tokyo, Japan) and homogenizing at 1,500 rpm for 2 minutes. The resulting homogenate was then incubated at 65°C for 10 minutes, followed by centrifugation at 12,000 × *g* for 2 minutes. Genomic DNA was obtained from the supernatant using the MPure-12 system and the MPure Bacterial DNA Extraction Kit (MP Biomedicals, Solon, OH, USA). The genome sequence was obtained through DNBSEQ-G400RS shotgun sequencing (MGI Tech, Shenzhen, China). The DNBseq library was prepared with the MGIEasy FS DNA Library Prep Set (MGI Tech). A total of 22,781,687 reads with 2 × 200-bp paired-end read lengths were obtained and then were trimmed and quality-filtered using Cutadapt (version 4.4) (6) and Sickle (version 1.33) (7), and *de novo* assembly was performed using SPAdes (version 3.15.5) (8). Contaminated contigs were detected and removed through the National Center for Biotechnology Information (NCBI) Contamination Screen, and contigs less than 200 bp in length were excluded. Genome completeness was assessed using BUSCO (version 5.3.2, ascomycota_odb10 data set) (9), revealing 98.1% completeness. All software was used with default settings unless otherwise indicated.

The draft genome sequence of strain TS-2 consisted of 2,189 scaffolds (40,665,975 bp with a scaffold *N*_50_ value of 870,479 bp, an average depth of 197.0×, and a G + C content of 49.5%), and a total of 14,164 protein-coding genes were predicted using BRAKER2 (version 2.1.6) (10). Among these genes, 14 genes were identified as putative laccase/multicopper oxidase encoding genes, which were considered to be responsible for Mn(II) oxidation (11, 12). These results suggested that Mn(II) oxidation in the genus *Periconia* may be attributed to the activities of these laccases.

## Data Availability

The genome sequence of strain TS-2 is available in NCBI GenBank under accession number GCA_030378425.1. The SRA raw sequence data are available as accession number SRX20546969, under BioProject number PRJNA977381 and BioSample number SAMN35523210.
